# Inducing cell death in Glioblastoma

**DOI:** 10.1186/1753-6561-9-S7-A24

**Published:** 2015-10-27

**Authors:** Shahed Tappuni, Janis Noonan, Brona Murphy

**Affiliations:** 1 Royal College of Surgeons in Ireland, Dublin, Ireland

## Background

Glioblastoma is the most common primary malignant brain tumor, and despite multimodal treatment with surgery, radiotherapy, and temozolomide (TMZ) chemotherapy, the prognosis is poor, with a median survival of 16 to 19 months [1]. Therefore, alternative treatments are necessary.

A therapy anticipated to be one of the most effective anti- cancer treatments is tumor necrosis factor-related apoptosis-inducing ligand (TRAIL) but resistance to TRAIL remains a significant challenge [2].

Like TRAIL, TMZ induces apoptosis; however, unlike TRAIL, TMZ also induces autophagy [3], which may hinder the apoptotic effects of the drug. Recently, the basal level of autophagy present in cells prior to treatment has been implicated as a potential indicator of treatment responsiveness/resistance. To assess the basal level of autophagy in a panel of glioblastoma cell lines in order to determine if these levels correspond to glioblastoma responsiveness and resistance to different treatment strategies.

## Methods

Fluorescence-activated cell sorting (FACS) and confocal imaging were utilized to assess the basal level of autophagy in the following GBM cells - MZ294, MZ304, MZ18, MZ51, MZ256, MZ327, U251, U343, U373, A172 and U87. MTT assays were utilized to measure cell viability following differing treatment combinations. Statistical analysis was performed using unpaired student t-tests. A *p* value <0.05 was considered statistically significant.

## Results

FACS analysis demonstrated that the TMZ-resistant cell lines MZ256 and MZ51 displayed the highest basal levels of autophagy. These results were supported by the confocal imaging experiments. The same cell lines also demonstrated resistance to TRAIL and to the combined treatment of TRAIL and TMZ.

Inhibition of autophagy in MZ18, MZ256, MZ51 and MZ304 with Bafilomycin A1 (Baf-A1) sensitized these cell lines to one or more of the treatment options. Specifically, MZ18, MZ256 and MZ51 have responded largely with the combination of TRAIL and Baf-A1 whereas MZ304 has responded better with the combination of TMZ and Baf-A1. But all of the four primary cell lines have a slightly higher response with the combination of TRAIL, TMZ and Baf-A1.

## Conclusion

The primary GBM cell lines display higher basal levels of autophagy than the commercial GBM cell lines, and are more resistant to treatment. Inhibiting autophagy in such cell lines resulted in a better response to treatment. These findings may help in the future treatment of glioblastoma.
